# Neuroclinical Framework for the Role of Stress in Addiction

**DOI:** 10.1177/2470547017698140

**Published:** 2017-04-10

**Authors:** Laura E. Kwako, George F. Koob

**Affiliations:** 1 National Institute on Alcohol Abuse and Alcoholism, National Institutes of Health, Bethesda, MD, USA

**Keywords:** Addiction, stress, neuroscience, negative affect, alcohol

## Abstract

Addiction has been conceptualized as a three-stage cycle—*binge/intoxication*, *withdrawal/negative affect*, and *preoccupation/anticipation*—that worsens over time and involves allostatic changes in hedonic function via changes in the brain reward and stress systems. Using the *withdrawal/negative affect* stage and negative reinforcement as an important source of motivation for compulsive drug seeking, we outline the neurobiology of the stress component of the *withdrawal/negative affect* stage and relate it to a derivative of the Research Domain Criteria research construct for the study of psychiatric disease, known as the Addictions Neuroclinical Assessment. Using the Addictions Neuroclinical Assessment, we outline five subdomains of negative emotional states that can be operationally measured in human laboratory settings and paralleled by animal models. We hypothesize that a focus on negative emotionality and stress is closely related to the acute neurobiological alterations that are experienced in addiction and may serve as a bridge to a reformulation of the addiction nosology to better capture individual differences in patients for whom the *withdrawal/negative affect* stage drives compulsive drug taking.

## Conceptual Framework

### What Is Stress?

Selye^1^ defined stress as responses to demands (usually noxious) upon the body that historically have been defined by various physiological changes that include activation of the hypothalamic–pituitary–adrenal (HPA) axis. However, a definition of stress that is more compatible with its many manifestations in the organism is “anything which causes an alteration of psychological homeostatic processes.”^2^ In fact, in a seminal paper, Mason^3^ argued the importance of psychological stress for eliciting a stress response, even among physical stressors and that many physical challenges absent psychological stress are not stressful.

The physiological response that is most associated with a state of stress is an elevation of glucocorticoids that derive from the adrenal cortex. This response is controlled by the HPA axis. Vale et al.^4^ first demonstrated that corticotropin-releasing factor (CRF) initiates the HPA axis neuroendocrine stress response (adrenocorticotropic hormone and ultimately glucocorticoids) by binding CRF_1_ receptors in the anterior pituitary after release into portal blood. CRF from the paraventricular nucleus of the hypothalamus was then identified as the primary controller in the HPA axis. Glucocorticoids function to increase and maintain blood sugar by elevating gluconeogenesis, and they decrease immune function by blocking proinflammatory proteins. These responses facilitate mobilization of the body in response to acute stressors. However, we now know that neurocircuits in the brain mediate behavioral responses to stressors and play a major role in “psychological homeostasis.”

Of relevance for this review, comorbidity between addictive and stress-related disorders is high. In the third wave of the National Epidemiologic Survey on Alcohol and Related Conditions (NESARC), the 12-month odds ratio for posttraumatic stress disorder (PTSD; i.e., the psychiatric disease most directly linked to stress exposure) and any substance use disorder was 1.3; the lifetime odds ratio was 1.5.^5^ Furthermore, in the National Comorbidity Survey-Replication, a diagnosis of PTSD at Time 1 was associated with odds ratios of 3.2 and 5.4 for alcohol and illicit drug dependence, respectively, at Time 2, 10 years later, among those individuals not substance dependent at Time 1.^6^

### What Is Addiction?

Addiction can be defined in many different ways, but one definition that has been generally adopted in the field is that addiction is a chronic, relapsing disorder that is characterized by a compulsion to seek and take drugs and the loss of control over drug intake. Others have emphasized a further characteristic, notably “the emergence of a negative emotional state (e.g., dysphoria, anxiety, and irritability) that defines a motivational withdrawal syndrome when access to the drug is prevented.”^7^ Indeed, some theorists have argued that such a negative emotional state is the defining feature of dependence on a drug:

The notion of dependence on a drug, object, role, activity or any other stimulus-source requires the crucial feature of negative affect experienced in its absence. The degree of dependence can be equated with the amount of this negative affect, which may range from mild discomfort to extreme distress, or it may be equated with the amount of difficulty or effort required to do without the drug, object, etc.^8^

Using such a framework, addiction has been conceptualized as a three-stage cycle—*binge/intoxication*, *withdrawal/negative affect*, and *preoccupation/anticipation*—that worsens over time and involves allostatic changes in hedonic function via changes in the brain reward and stress systems. Allostasis is defined as stability through change via a feed-forward mechanism that readjusts parameters to a new hedonic set point but outside the homeostatic range. Two primary sources of reinforcement—positive and negative reinforcement—have been hypothesized to play a role in this allostatic process. Positive reinforcement is defined as the process by which the presentation of a stimulus increases the probability of a response. Negative reinforcement is defined as the process by which the removal of an aversive stimulus (or aversive state, in the case of addiction) increases the probability of a response.

Another framework with which to conceptualize drug addiction is the impulsivity–compulsivity continuum, in which *impulsivity* can be behaviorally defined as “actions which are poorly conceived, prematurely expressed, unduly risky, or inappropriate to the situation and that often result in undesirable consequences.”^9^ Impulsivity is a core deficit in substance abuse disorders.^10^ It can be measured in multiple ways, but two domains dominate: the choice of a smaller, immediate reward over a larger, delayed reward^11^ or the inability to inhibit behavior by changing the course of action or to stop a response once it is initiated.^12^ Operationally, delay-to-gratification tasks (e.g., delayed discounting tasks, impulsive choice) and the Stop-Signal or Go/No-Go task (behavioral impulsivity) have both been used as measures of the various domains of impulsivity.^13,14^

In contrast, “compulsivity can be characterized by perseverative, repetitive actions that are excessive and inappropriate to a situation.”^15^ Individuals who suffer from compulsions often recognize that the behaviors are harmful, but they nonetheless feel emotionally compelled to perform them. Performance of these behaviors reduces tension, stress, or anxiety.^15,16^ Operationally, in animal models, responding for a drug or alcohol in the face of adverse consequences^17^ or responding for a drug or alcohol on a progressive-ratio schedule of reinforcement^18^ has been argued to reflect compulsivity. Thus, in addition to the positive reinforcement associated with high impulsivity linked to the early stages of the addiction process, an additional source of motivation is recruited, namely negative reinforcement.

**Figure 1.**
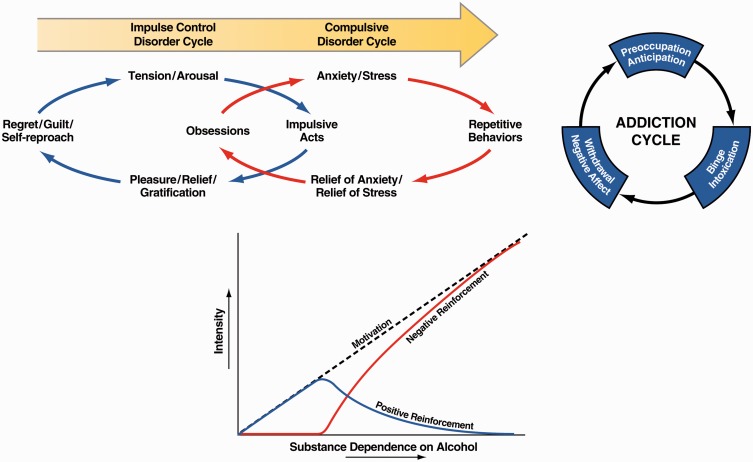
(Top left) Diagram showing the stages of impulse control disorder and compulsive disorder cycles related to the sources of reinforcement. In impulse control disorders, an increasing tension and arousal occurs before the impulsive act, with pleasure, gratification, or relief during the act. Following the act, there may or may not be regret or guilt. In compulsive disorders, there are recurrent and persistent thoughts (obsessions) that cause marked anxiety and stress followed by repetitive behaviors (compulsions) that are aimed at preventing or reducing distress.^19^ Positive reinforcement (pleasure/gratification) is more closely associated with impulse control disorders. Negative reinforcement (relief of anxiety or relief of stress) is more closely associated with compulsive disorders (taken with permission from Koob^20^). (Top right) Collapsing the cycles of impulsivity and compulsivity results in the addiction cycle, conceptualized as three major components: *preoccupation/anticipation*, *binge/intoxication*, and *withdrawal/negative affect* [taken with permission from Koob^22^). (Bottom) Change in the relative contribution of positive and negative reinforcement constructs during the development of substance dependence on alcohol (taken with permission from Koob^20^).

This impulsivity–compulsivity continuum has a nosological history. Subjects with classic atypical impulse control disorders, such as kleptomania, experience an increasing sense of tension or arousal before committing an impulsive act; pleasure, gratification, or relief at the time of committing the act; and regret, self-reproach, or guilt following the act.^19^ In contrast, subjects with classic compulsive-like disorders, such as obsessive–compulsive disorder, experience anxiety and stress before committing a compulsive repetitive behavior and relief from the stress by performing the compulsive behavior.^19^ We have argued that drug addiction progresses from a source of positive reinforcement that may indeed involve more elements of impulsivity to a source of negative reinforcement that may involve more elements of compulsivity (Figure 1).^20^ The three-stage cycle of addiction, with the embedded conceptual sources of motivation of positive and negative reinforcement that parallel impulsivity and compulsivity (Figure 1), are not unique to drug addiction and generalize to non-drug or “process” addictions. In a recent review,^21^ the authors identified three major domains of neurofunctional impairment related to gambling disorder, namely the loss of control, craving/withdrawal, and the neglect of other areas of life. These domains closely parallel the domains outlined in the three stages of the addiction cycle and the Addictions Neuroclinical Assessment (ANA) framework (see below).

### Neurobiology of Stress

Key highly conserved responses to stressors in the environment comprise fight or flight. A superstructure in the basal forebrain, the extended amygdala, processes fear, threats, and anxiety in humans (i.e., fight or flight responses)^7,23^ and engages the neurocircuitry of negative emotional states. The extended amygdala shares similarities in morphology, neurochemistry, and connectivity and is composed of the central nucleus of the amygdala (CeA), bed nucleus of the stria terminalis (BNST), and a transition zone in the posterior medial part (shell) of the nucleus accumbens (NAcSh).^24^ The extended amygdala receives inputs from various regions of the brain that are involved in emotion, but most importantly the prefrontal cortex. The extended amygdala projects heavily to the hypothalamus and other midbrain structures that are involved in the expression of emotional responses.^24,25^ When animals are exposed to a stressor, they exhibit an enhanced freezing response to a conditioned fear stimulus, an enhanced startle response to a startle stimulus, the avoidance of open areas, open arms, and heights, and enhanced species-typical responses to an aversive stimulus. All of these responses are at least partially mediated by the extended amygdala. In psychopathology, dysregulation of the extended amygdala has been hypothesized to play a key role in disorders that are related to stress and negative emotional states, such as PTSD, general anxiety disorder, phobias, affective disorders, and addiction.^26,27^

Two neurochemical systems, CRF and dynorphin, play a key role in the extended amygdala to effect such behavioral changes. Both are also implicated in the psychopathology associated with the extended amygdala, and both are the focus of individual differences in stress pathology. The glucocorticoid response mobilizes the body for physiological responses to stressors; CRF plays another role by mobilizing the body’s behavioral response to stressors via brain circuits outside the hypothalamus. In an early study, CRF was intracerebroventricularly injected into the brain in naive rats, which produced hyperactivity and hyperarousal in a familiar environment but a very pronounced freezing-like response in a novel stressful environment.^28^ Subsequent work showed that a prominent system that mediates such responses to CRF and fear and anxiety in general is the extended amygdala. The administration of competitive CRF receptor antagonists was shown to have opposite anti-stress effects. This observation was critical because it confirmed a role for endogenous CRF in behavioral responses to stressors (for review, see Koob and Zorrilla^29^).

The dynorphin-κ opioid system also plays a key role in affecting behavioral responses to stressors. Dynorphins contain the leucine (leu)-enkephalin sequence at the *N*-terminal portion of the molecule and are endogenous ligands for the κ opioid receptor.^30^ Dynorphins are widely distributed in the central nervous system^31^ and play a role in neuroendocrine regulation, pain regulation, motor activity, cardiovascular function, respiration, temperature regulation, feeding behavior, and stress responsivity. Dynorphins produce aversive dysphoric-like effects in animals and humans and have been hypothesized to mediate behavioral responses to stressors and negative emotional states (for review, see Van’t Veer and Carlezon^32^).

**Figure 2.**
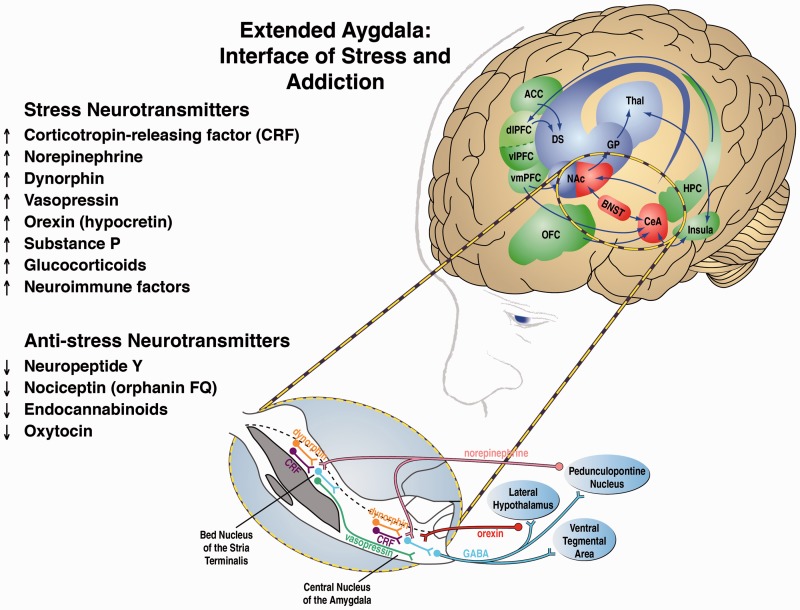
Neural circuitry associated with the three stages of the addiction cycle, with a focus on the *withdrawal/negative affect* stage and extended amygdala. The targets identified in this review that are relevant to the *withdrawal/negative affect* stage are listed on the left. On the right is the neurocircuitry of the pathophysiology of addiction. *Binge/intoxication* stage (blue): Drugs may engage associative mechanisms and reward neurotransmitters (such as dopamine and opioid peptides) in the nucleus accumbens shell and core (incentive salience, defined as a motivational response of the brain to reward-predicting stimuli) and then engage stimulus-response habits that depend on the dorsal striatum. *Withdrawal/negative affect* stage (red): The negative emotional state of withdrawal engages activation of the extended amygdala. The extended amygdala is composed of several basal forebrain structures, including the bed nucleus of the stria terminalis, central nucleus of the amygdala, and a transition zone in the medial portion (or shell) of the nucleus accumbens. Neurotransmitter systems engaged in the neurocircuitry of the extended amygdala that convey negative emotional states are indicated by upward-pointing arrows, and neurotransmitter systems that may buffer negative emotional states are indicated by downward-pointing arrows. *Preoccupation/anticipation (craving)* stage (green): This stage involves the prefrontal cortex and includes representations of contingencies, representations of outcomes, and executive function. An important neurotransmitter that is engaged in craving responses is glutamate. The magnified section (blue oval) illustrates the extended amygdala in detail. A major neurotransmitter in the extended amygdala is CRF, which projects to the brainstem where noradrenergic neurons provide a major projection reciprocally to the extended amygdala. Green/blue arrows indicate glutamatergic projections. Acb, nucleus accumbens; ACC, anterior cingulate cortex; BLA, basolateral amygdala; BNST, bed nucleus of the stria terminalis; CeA, central nucleus of the amygdala; CRF, corticotropin-releasing factor; DGP, dorsal globus pallidus; dlPFC, dorsolateral prefrontal cortex; NE, norepinephrine; OFC, orbitofrontal cortex; SNc, substantia nigra pars compacta; VGP, ventral globus pallidus; vlPFC and vmPFC, ventral prefrontal cortex; VTA, ventral tegmental area (modified with permission from Koob and Volkow^34^; see also Koob^33^ and Koob and Mason^35^).

Other key neurotransmitter systems, all of which interact with the extended amygdala, that mediate behavioral responses to stressors include norepinephrine, vasopressin, hypocretin (orexin), substance P, proinflammatory cytokines, and key neurotransmitter systems that act in opposition to the brain stress systems, such as neuropeptide Y (NPY), nociceptin, and endocannabinoids. Altogether, these neurochemical systems set the tone and modulate emotional expression, particularly negative emotional states, via the extended amygdala (Figure 2).^33^ These stress systems and their relevance for addiction are comprehensively reviewed in Koob.^22^

## Neurobiology of Addiction

The neurobiological basis of the *binge/intoxication* stage of the addiction cycle involves the activation of reward circuits and facilitation of incentive salience circuits. Drugs of abuse are rewarding but also confer motivational properties to previously neutral stimuli, a process known as incentive salience. Drug reward and drug-induced incentive salience are mediated largely by neurocircuitry in the basal ganglia. For most of the major drugs of abuse, animal studies have shown that their reinforcing actions are mediated by the release of dopamine and opioid peptides in the ventral striatum (NAc).^34^ Human imaging studies have shown that intoxicating doses of most drugs of abuse and alcohol release dopamine and opioid peptides into the ventral striatum.^36,37^ Activation of the ventral striatum leads to the recruitment of basal ganglia–globus pallidus–thalamic–cortical loops that engage the dorsal striatum in habit formation and habit strengthening that is hypothesized to be the beginning of compulsive-like responding for drugs.^38^

In the *withdrawal/negative affect* stage, two processes, possibly acting in parallel, are hypothesized to form the neurobiological basis for the loss of function in the reward systems (within-system neuroadaptation) in the ventral striatum and the recruitment of the brain stress systems (between-system neuroadaptation) in the extended amygdala.^39^ A within-system neuroadaptation was defined as the process by which the primary cellular response element to the drug (circuit A, reward circuit) adapts to neutralize the drug’s effects and have drug-opposite effects. Examples of within-system changes have been hypothesized to be molecular cellular changes within the reward circuits that are overactivated in the *binge/intoxication* stage and include the perturbations of intracellular signal transduction pathways, including changes in G-protein functioning and protein kinase A (PKA) activity and such transcription factors as cyclic adenosine monophosphate response element binding protein (CREB), and downstream ΔFosB, nuclear factor κB, and CDK5 that can modify gene expression.^40^ As dependence (defined as the manifestation of motivational withdrawal symptoms; i.e., elements of negative emotional states) develops, brain stress systems, such as CRF, norepinephrine, dynorphin, hypocretin, and substance P, are recruited, producing aversive or stress-like states.^41,42^ A between-system neuroadaptation was defined as a circuitry change in a circuit that is not circuit A, in which circuit B (stress circuit) may be triggered by activity in circuit A (i.e., the reward circuit). Within-system neuroadaptations can dynamically interact with between-system neuroadaptations, in which circuit B (i.e., the stress circuit) is activated either in parallel to affect a negative emotional state or in series to suppress the activity of circuit A to affect a negative emotional state.^33^ The CRF systems described above are recruited during repeated binge and withdrawal, with activation of the HPA axis, which, in turn, releases glucocorticoids, which, in turn, feed back to sensitize CRF systems in the extended amygdala that activate circuitry to drive negative emotional states (Table 1).^33,43,44^ The dynorphin-κ system has long been hypothesized to mediate negative emotional states of drug withdrawal by suppressing activity of the mesocorticolimbic dopamine system.^42^ Data to date suggest that these actions may be mediated by dynorphin activity in the NAcSh.^45,46^ The dynorphin-κ system may also interact with the CeA and be involved in promoting anxiety-like responses.^47^ In parallel, as noted above, there are anti-stress buffer systems in the extended amygdala that have the opposite effects to the stress-promoting modulatory systems. These include NPY, nociceptin, and endocannabinoids (Table 1). For example, NPY activation in the CeA has opposite effects to CRF.^48^ NPY blocks high compulsive-like alcohol administration, blocks the transition to excessive drinking with the development of dependence, and blocks the increase in γ-aminobutyric acid (GABA) release in the CeA that is produced by alcohol.^49,50^ The combination of decreases in reward neurotransmitter function and the recruitment of brain stress systems provides powerful motivation for reengaging in drug taking and drug seeking.

**Table 1.** Molecular neurocircuits of the *withdrawal/negative affect* stage as focal points for neuroplasticity in addiction.

Circuit	Neurotrasmitter	Modulatory response	References^a^
Decreased reward neurotransmitters		
VTA–NAc	CRF	↑	Grieder et al.^51^
Habenula–VTA	Acetylcholine	↓	Fowler et al.^52^
Increased stress neurotransmitters		
CeA	CRF	↑	Funk et al.^53^
Brainstem–BNST	Norepinephrine	↑	Delfs et al.^54^
Hypothalamus–CeA	Hypocretin	↑	Schmeichel et al.^55^
CeA	Substance P	↑	Barbier et al.^56^
NAc shell	Dynorphin	↑	Carlezon et al.^42^
CeA	Dynorphin	↑	Kallupi et al.^47^
PVN/SON–CeA	Vasopressin	↑	Hernandez et al.^57^
Decreased anti-stress neurotransmitters		
ARC–CeA	Neuropeptide Y	↓	Heilig and Thorsell^48^
CeA	Nociceptin	↓	Economidou et al.^58^
CeA	Endocannabinoids	↓	Sidhpura and Parsons^59^

ARC: arcuate nucleus; CeA: central nucleus of the amygdala; NAc: nucleus accumbens; PVN: paraventricular nucleus; SON: supraoptic nucleus; VTA: ventral tegmental area.

a References are key papers that show either direct evidence of the circuit outlined or hypothesize the existence of such modulation. The second column (Circuit) indicates either a neurotransmitter circuit or, where only one neuroanatomical site is listed, a local circuit. Arrows represent the direction of modulation.

Thus, multiple circuits that involve multiple modulatory neurotransmitter systems converge on the extended amygdala to mediate negative emotional states associated with the *withdrawal/negative affect* stage. Each theoretically conveys differential qualitative dimensions to the construct of a negative emotional state that forms a basis for the dimensions of a neuroclinical assessment for the *withdrawal/negative affect* stage of the addiction cycle (Table 1).

The *preoccupation/anticipation* (“craving”) stage mediates the impairment of executive control in addiction via prefrontal cortex circuits. Executive function can be defined as an overall control circuit that limits impulsive and compulsive responses, delays reinforcement, and makes appropriate choices and responses, among others. Two systems have been conceptualized: a Go system and a Stop system, which do not necessarily act in opposition.^44^ The Go system consists of parts of the anterior cingulate cortex, dorsal prefrontal cortex, and orbitofrontal cortex and engages habits via the basal ganglia. The Stop system consists of the ventral prefrontal cortex, orbitofrontal cortex, and other prefrontal regions that overlap with the Go system. Critically, Stop system projections inhibit the basal ganglia incentive salience system and extended amygdala stress system. In individuals with substance use disorders, there are disruptions of decision making, impairments in the maintenance of spatial information, and impairments in behavioral inhibition, all of which can drive craving and drug seeking. Craving, defined as the desire for a drug or alcohol in the absence of the drug, has been hypothesized to be divided into two domains: reward craving (drug seeking induced by drugs or stimuli linked to drugs) and relief craving (drug seeking induced by an acute stressor or a state of stress).^60^ The brain circuitry that mediates both of these constructs can parallel the hypothesized subcortical dysregulations associated with the *binge/intoxication* and *withdrawal negative/affect* stages and can contribute to relapse during protracted abstinence in the *preoccupation/anticipation* (“craving”) stage.

## Neuroclinical Assessment: From Reward to Stress and Back

The nosological research framework termed Research Domain Criteria (RDoC) originated as part of the National Institute of Mental Health (NIMH) 2008 strategic plan, with the goal of creating a research framework for studying psychiatric disorders. The NIMH framework was conceptually grounded in neuroscience research and spanned five domains: Negative Valence Systems, Positive Valence Systems, Cognitive Systems, Systems for Social Processes, and Arousal and Regulatory Systems. RDoC domains are organized by units of analysis, ranging from genes to paradigms (for an overview of the RDoC matrix, see http://www.nimh.nih.gov/research-priorities/rdoc/research-domain-criteria-matrix.shtml; accessed 20 January 2017), and this approach has generated much conceptual and methodological discussion.^61–65^ We have proposed a more parochial, within-disorder, research approach, the Addictions Neuroclinical Assessment (ANA) framework,^66^ which captures information in three of the five original RDoC domains.

The ANA domains were derived from the conceptual framework outlined above, in which drug addiction derives from a three-stage cycle with conceptual roots in impulsivity and compulsivity, the recruitment of positive and negative reinforcement, and interactions between the neurobiological substrates of reward and stress. Three functional domains—executive function, incentive salience, and negative emotionality—were proposed as described above. The *withdrawal/negative affect* stage of this cycle, including stress and negative emotional states but not limited to withdrawal and representing the negative emotionality domain, is the focus of the discussion that follows.^67^

### Negative Emotionality

Although often not emphasized, the reports of individuals who suffer from drug addiction are replete with descriptions of overall self-reported dysphoria and various manifestations of negative emotional states.^68,69^ Such descriptions include depression, anxiety, anhedonia, dysphoria, malaise, alexithymia, hyperkatifeia, emotional pain, physical pain, irritability, and sleep disturbances. A self-medication hypothesis has long infiltrated theories of addiction but has been dismissed, usually based on the grounds that both humans and animals will self-administer drugs without undergoing physical withdrawal. However, a rather common misunderstanding of tolerance and withdrawal in addiction is that they represent purely “physical” phenomena,^70–73^ rather than motivational constructs. Indeed, both tolerance (defined as increased reward seeking and taking more drug to produce the same effect)^74^ and withdrawal (defined as a motivational withdrawal syndrome characterized by dysphoria, anxiety, and irritability when the reward that is sought is unavailable)^67,75^ are present in all drug and behavioral addictions.^76,77^ For example, a complete assessment of reward constructs must include measurements of hypohedonia.^78^ Hypohedonia is widely documented as a clinical feature of addiction^79–83^ and is highly associated with increased craving for drugs of abuse^84^ and relapse.^85^

### Opponent Process as a Guiding Principle

**Figure 3.**
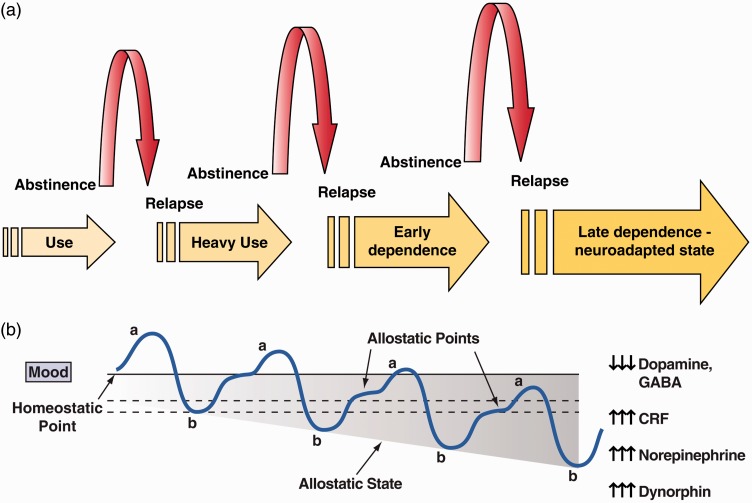
(a) Schematic of the progression of drug and alcohol dependence over time, illustrating the shift in underlying motivational mechanisms. From initial, positive-reinforcing, pleasurable effects of drugs and alcohol, the addiction process progresses over time to being maintained by negative-reinforcing relief from a negative emotional state. Neuroadaptations that encompass the recruitment of extrahypothalamic CRF systems are key to this shift (taken with permission from Heilig and Koob^88^). (b) The *a-process* represents a positive hedonic or positive mood state, and the *b-process* represents the negative hedonic or negative mood state. The affective stimulus (state) has been argued to be the sum of both the *a-process* and the *b-process*. An individual who experiences a positive hedonic mood state from a drug of abuse with sufficient time between re-administering the drug is hypothesized to retain the *a-process*. An appropriate counteradaptive opponent process (*b-process*) that balances the activational process (*a-process*) does not lead to an allostatic state. The changes in the affective stimulus (state) in an individual with repeated frequent drug use may represent a transition to an allostatic state in the brain reward systems and, by extrapolation, a transition to addiction (see text). Notice that the apparent *b-process* never returns to the original homeostatic level before drug taking begins again, thus creating a greater and greater allostatic state in the brain reward system. The counteradaptive opponent-process (*b-process*) does not balance the activational process (*a-process*) but in fact shows a residual hysteresis. Although these changes that are illustrated in the figure are exaggerated and condensed over time, the hypothesis is that even during post-detoxification (a period of “protracted abstinence”), the reward system still bears allostatic changes. The following definitions apply: *allostasis*, the process of achieving stability through change; *allostatic state*, a state of chronic deviation of the regulatory system from its normal (homeostatic) operating level; *allostatic load*, the cost to the brain and body of the deviation, accumulating over time, and reflecting in many cases pathological states and accumulation of damage (Modified with permission from Koob and Le Moal^41^).

The interaction between reward and stress is dynamic both phenotypically and neurobiologically. Low levels of acute stress have long been considered rewarding. Glucocorticoids have rewarding properties and can even be self-administered by animals.^86^ However, chronic stress generally leads to malaise, irritability, and dysphoria, which drive mechanisms of negative reinforcement. Neurobiologically, accumulating evidence links excessive activation of the reward system as a causal mechanism for activation of the brain stress systems (see below). In the domain of motivation in addiction, the interaction between reward and stress was inextricably linked with hedonic, affective, or emotional states in the context of temporal dynamics by the opponent-process theory of motivation.^87^ Here, hedonic, affective, or emotional states, once initiated, are automatically modulated by mechanisms that reduce the intensity of hedonic feelings, presumably mediated by the central nervous system. Solomon and Corbit argued that there are affective or hedonic habituation (or tolerance) systems and affective or hedonic withdrawal (abstinence) systems. They defined two processes: the *a-process* and *b-process*. The *a-process* consists of either positive or negative hedonic responses. In the case of addiction, one would hypothesize that the *a-process* is a positive hedonic response to administration of a highly rewarding drug. The *a-process* occurs shortly after the presentation of a stimulus, correlates closely with the stimulus intensity, quality, and duration of the reinforcer, and shows tolerance. In contrast, the *b-process* appears after the *a-process* has terminated, is sluggish in onset, is slow to build up to an asymptote, is slow to decay, and gets larger with repeated exposure (Figure 3). The *b-process* would be the beginning of the development of the negative emotional state associated with the *withdrawal/negative affect* stage.

Such an opponent process has been demonstrated in animals.^89^ In an early study, chronic binge-like cocaine self-administration resulted in an opposite effect on brain stimulation reward thresholds (i.e., a measure of hedonic activity in the brain), namely an elevation of brain-stimulation reward thresholds.^89^ Subsequent studies showed that the elevation of brain reward thresholds that was associated with withdrawal from chronic administration of drugs of abuse is a common element of all drugs of abuse, including cocaine,^89^ amphetamine,^90^ opioids,^91^ cannabinoids,^92^ nicotine,^93^ and alcohol.^94^ A series of studies revealed elevations of brain reward thresholds during withdrawal in animal models. Key neuropharmacological evidence has been generated that shows that both reversing reward deficit neurotransmission and reversing stress surfeit neurotransmission can block the elevation of reward thresholds produced by drug withdrawal.^95^

A key component that drives negative emotional states in general and hypohedonia in particular and is associated with the *withdrawal/negative affect* stage of the addiction cycle is engagement of the brain stress systems, including both the HPA and extrahypothalamic systems.^96^ As noted above, the brain stress systems include such neurotransmitter systems as CRF, dynorphin, norepinephrine, hypocretin (orexin), substance P, and vasopressin. Equally compelling is evidence of the dysregulation of brain anti-stress systems, such as NPY, nociceptin, endocannabinoids, and oxytocin. Increased activity in brain stress systems and decreased activity in brain anti-stress systems are hypothesized to significantly contribute to negative emotionality.^96^

### Neuroclinical Assessment: Anhedonia, Hypohedonia, and Dysphoria

The neurocircuitry of anhedonia, hypohedonia, and dysphoria to a large extent has been hypothesized to reflect “within-system” changes in the mesocorticolimbic dopamine system or opioid peptide systems the converge on the NAc. “Between-system” changes that mediate anhedonia, hypohedonia, and dysphoria include the activation of neurocircuits that are involved in stress (CRF in the CeA and BNST) or neurocircuits that feed back to suppress dopaminergic activity (CRF and/or dynorphin or acetylcholine in the VTA, NAc, and habenula; Table 1). Animal models with construct validity for anhedonia, hypohedonia, and dysphoric-like responding that have helped elucidate the respective neurocircuits include measures of brain stimulation reward thresholds (intracranial self-stimulation), sucrose preference, progressive-ratio responding, and the probabilistic reward task in animals (Table 2). Human laboratory assessments of anhedonia, hypohedonia, and dysphoria range from standard self-report measures, such as the Beck Depression Inventory and Hamilton Anxiety Rating Scale, to measures that focus selectively on negative reward constructs, such as the Fawcett-Clark Pleasure Scale (Table 3). More operational measures of anhedonia, hypohedonia, and dysphoria include the probabilistic reward task^97–100^ and Effort for Expenditure for Rewards Task,^101^ among others.

**Table 2.** Animal models for negative emotional states.

Negative emotionality				
Assessment	Measure	References	Repeated testing within subject	Animal model
Animal Model: anhedonia, hypohedonia, and dysphoria	Intracranial self-stimulation	Markou and Koob^89^	Yes	Rat
	Conditioned place aversion	Hand et al.^102^	No	Rat
	Disrupted operant responding	Gellert and Sparber^103^	Yes	Rat
	Drug discrimination	Gauvin and Holloway^104^	Yes	Rat
	Sucrose preference	Hammami-Abrand Abadi et al.^105^	Yes	Rat, mouse
	Probabilistic reward task	Pizzagalli et al.^99^	Yes	Rat
Neuroclinical: anxiety, stress reactivity, and irritability	Progressive-ratio responding	Wee et al.^106^	Yes	Rat, mouse
	Elevated plus maze	Fawcett et al.^107^	No	Rat, mouse
	Defensive burying	Bagby et al.^108^	No	Rat, mouse
	Light/dark box	Bourin and Hascoet^109^	No	Rat, mouse
	Marble burying task	Njung’e and Handley^110^	No	Rat, mouse
Neuroclinical: pain and hyperkatifeia	Mechanosensitivity pain test	Edwards et al.^111^	Yes	Rat, mouse
	Thermal pain hypersensitivity (tail-flick test)	Raghavendra et al.^112^	Yes	Rat, mouse
Neuroclinical: malaise, sleep disturbances, and arousal	Electroencephalogram sleep measures (EEG activity)	Walker and Zornetzer^113^	Yes	Rat, mouse
	Locomotor activity	Pulvirenti and Koob^114^	Yes	Rat, mouse

EEG: electroencephalogram.

**Table 3.** Human laboratory tests for negative emotional states.

Negative emotionality				
Negative emotional states	Measure	Reference	Time to complete	Type of task
Neuroclinical: anhedonia, hypohedonia, and dysphoria	Approach Avoidance Task	Heuer et al.^115^	10 min	Behavioral
	Two-step Task (model-free model-based)	Sebold et al.^116^	15 min	Behavioral
	Beck Depression Inventory	Beck et al.^117^	5 min	Self-report
	Fawcett-Clark Pleasure Scale	Fawcett et al.^107^	5 min	Self-report
Neuroclinical: anxiety, stress reactivity, and irritability	Cyberball	Williams and Jarvis^118^	10 min	Behavioral
	Trier Social Stress Test	Kirschbaum et al.^119^	20 min	Behavioral
	Buss-Durkee Hostility Inventory	Buss and Durkee^120^	15 min	Self-report
Neuroclinical: pain and hyperkatifeia	Cold Pressor Task	Lovallo^121^	10 min	Behavioral
	Toronto Alexithymia Scale	Bagby et al.^108^	5 min	Self-report
	Facial Emotion Matching Task	Hariri et al.^122^	10 min	Neuroimaging
Neuroclinical: malaise, sleep disturbances, and arousal	Malaise Inventory	Rodgers et al.^123^	5 min	Self-report
	Pittsburgh Sleep Quality Index	Buysse et al.^124^	5 min	Self-report
	Behavioral Activation System	Carver and White^125^	5 min	Self-report

### Neuroclinical Assessment: Anxiety, Stress Reactivity, and Irritability

The neurocircuitry of anxiety, stress, and irritability are hypothesized to involve “between-system” changes that include activation of neurocircuits involved in stress (CRF, norepinephrine, vasopressin, and hypocretin in the CeA and BNST; Table 1). Animal models with construct validity for anxiety-like behavior, stress reactivity, and irritability-like behavior that have helped elucidate the neurocircuitry associated with anxiety, stress, and irritability include the elevated plus maze, defensive withdrawal test, defensive burying test, marble burying test, and social interaction test (Table 2). Human laboratory assessments of anxiety, stress reactivity, and irritability range from standard self-report measures, such as the Beck Anxiety Inventory and Hamilton Depression Rating Scale, to those that focus selectively on trauma constructs, such as the Childhood Trauma Questionnaire (Table 3). More operational measures of anxiety, stress reactivity, and irritability include the Cyberball Test, Trier Social Stress Test, and Buss-Durkee Hostility Inventory (Table 3).

### Neuroclinical Assessment: Pain and Hyperkatifeia

The neurocircuitry of pain and analgesia are hypothesized to involve “between-system” changes that include the activation of pain circuits and also neurocircuits that are involved in stress (CRF, norepinephrine, vasopressin, and substance P in the CeA and BNST; Table 1). Animal models with construct validity for pain and hyperalgesia that have helped elucidate the neurocircuitry associated with pain and the interaction between pain and stress include the hot plate test, tail flick test, and von Frey test (Table 2).

Both hyperalgesia and hyperkatifeia have been observed in humans during withdrawal from opioids and alcohol.^126,127^ Hyperalgesia can be defined as an increased sensitivity to pain. Hyperkatifeia (derived from the Greek word *katifeia* for dejection, sadness, or negative emotional state) is defined as the increased intensity of negative emotional/motivational symptoms and signs.^128^ Human laboratory assessments of hyperalgesia range from standard test batteries of pain thresholds to thermal, electrical stimulation, or pressure pain. More general tests of emotional liability include the Toronto Alexithymia Scale (Table 3). More operational measures of pain include tests that focus selectively on hyperalgesia (e.g., cold pressor test) and hyperkatifeia (e.g., Facial Emotion Matching Task; Table 3).

### Neuroclinical Assessment: Malaise, Sleep Disturbances, and Arousal

The neurocircuitry of malaise, sleep disturbances, and arousal are hypothesized to involve both “within-system” changes in the mesocorticolimbic dopamine system for arousal and malaise, but also “between-system” changes in neurocircuits that are involved in malaise (CRF, norepinephrine, vasopressin, and hypocretin in the CeA and BNST) and sleep/arousal (hypocretin in the hypothalamus; Table 1). Indeed, hypocretin (orexin) has been shown to play a critical role not only in addiction, as described above, but also in regulating arousal and coordinating the alertness that is necessary to pursue goal-directed behaviors.^129^ Animal models with construct validity for malaise, sleep disturbances, and arousal that have helped elucidate the neurocircuitry associated with these constructs in humans include activity measures, electroencephalography, and observations of peripheral physiological arousal. Patients who are addicted to various agents have present self-reported malaise,^130^ sleep disturbances,^131,132^ and disruptions in arousal.^133^ Malaise may be defined as an undefined sense of illness or unease without a specific cause. Within addictive disorders, sleep disturbances often take the form of insomnia and changes in sleep architecture.^131,132^ Dysregulated arousal may appear as hyperarousal in response to stressful stimuli or drug cues compared with individuals who are not addicted.^133^ Relatedly, hyperarousal is a key diagnostic criterion for PTSD, which is highly comorbid with addiction to various substances.^134^ Human laboratory assessments of these constructs include polysomnography for the evaluation of sleep and electroencephalography and peripheral signals (e.g., galvanic skin response, respiration, and heart rate) for the evaluation of arousal, in addition to self-report measures, such as the Malaise Inventory, Pittsburgh Sleep Quality Index, and Behavioral Activation System Scale (Table 3).

## Implications for Nosology of Addiction

Over time, the nosology of addictions has remained relatively static. The most recent iteration of the *Diagnostic and Statistical Manual of Mental Disorders* (DSM-5)^135^ combines the previous substance abuse and dependence categories into one, labeled Substance Use Disorder. This change also affords an assessment of disease severity based on symptom counts. Regardless, several problems exist with the current nosology, which may be addressed through the ANA and a focus on negative emotionality and stress. First, most of the specific diagnostic criteria load onto the same factor, despite the fact that in practice, considerable within-diagnosis heterogeneity exists and is a limiting factor in treatment outcome. Second, these criteria are largely not based on the neurobiology of addiction but rather on patient-reported symptoms. While patients’ who present complaints are a critical piece of diagnosis and treatment plan formulation, they are also insufficient for these tasks. For example, a patient who suffers from a particular form of cancer may complain of pain and fatigue; these presenting concerns, while important, do not form the basis of diagnosis. Instead, a diagnosis of cancer is made by considering alterations in patients’ biological systems, such as the presence of a tumor or an increase in cancer cells in the blood stream, which are diagnosed by imaging and/or blood tests. Currently, the presentation in subjects of the current diagnostic criteria of hedonic tolerance and motivational withdrawal (defined above) are most closely related to the actual neurobiological alterations that occur in addictions and may serve as a bridge to a reformulation of the nosology of addiction.

Even without a specific and definitive neurobiological marker, an emphasis on stress and negative affective states in addictive disorders, as discussed herein, could lead to the inclusion of these in future iterations of addiction diagnoses. For example, specifying whether an individual experiences significant dysphoria or relief craving during withdrawal, while still being symptom-based, would be one step closer toward a neurobiologically informed addiction diagnosis. It would also critically allow clinicians to identify treatments that would more closely align with a specific subtype of addiction. Overall, a strong emphasis on negative affective states that are associated with addiction could further the integration of neurobiology into the addiction nosology and improve treatment outcome. Given the significant public health costs associated with addictions, these improvements would be well worth the time and effort to further explore the role of stress and negative affect in addictions.
